# “Those Who Need It the Most”: Equity Framing in 2021 U.S. Print News About Food Assistance

**DOI:** 10.1089/heq.2023.0016

**Published:** 2023-08-10

**Authors:** Hina Mahmood, Pamela Mejia, Sarah B. Perez-Sanz, Kim Garcia, Sarah Gollust, Jeff Niederdeppe, Erika Fowler, Laura Baum, Breeze Floyd, Lori E. Dorfman

**Affiliations:** ^1^ Berkeley Media Studies Group, Public Health Institute, Oakland, California, USA.; ^2^ Division of Health Policy and Management, University of Minnesota, Minneapolis, Minnesota, USA.; ^3^ College of Agriculture and Life Sciences, Cornell University, Ithaca, New York, USA.; ^4^ Government Department, Wesleyan University, Middletown, Connecticut, USA.

**Keywords:** public health, racial inequity, food assistance, media, safety net programs, print news

## Abstract

**Objective:**

To understand how equity appeared in news about food assistance from 2021.

**Methods:**

We assessed a national sample of news articles (*N*=298) for equity arguments and language about racial and health equity.

**Results:**

Only 28% of coverage argued that food assistance programs promote equity. Just 6% mentioned people of color or named racial disparities in food access.

**Discussion:**

Narratives that explain how food assistance programs reduce inequities could deepen their policy appeal and broaden public perceptions around recipients.

**Health Equity Implications:**

There are opportunities for news coverage to expand the discussion of how food assistance programs improve racial and health equity outcomes.

## Introduction

Food assistance has been part of the U.S. social safety net for decades, although not without controversy over who should receive benefits and in what form. These issues became even more significant with the onset of the COVID-19 pandemic, when many Americans relied on food assistance. News coverage both reflects public dialogue and sets the agenda for what the public and policymakers think about, how they think about different issues, and the policy solutions they support.^1,2^ News can also shape public opinion, which can mobilize policymakers to respond and to implement policies that can impact health outcomes.^3^

In news from 2020, we found a narrative that evoked empathy for those who received food assistance during the pandemic, and emphasized that the government should play a larger role in addressing food insecurity.^4^ The news also highlighted the growing need for changes in programmatic design to ease access amid increasing institutional stress.

We did not explicitly look for equity arguments in the news in our first analysis, although food assistance programs are a key mechanism for reducing and ultimately eliminating disparities in health and health determinants that adversely affect excluded or marginalized groups.^5^ Mapping the discourse around the inequities that continue to underlie food assistance programs is an important part of understanding the evolving national policy discussion as the pandemic ends.

We wanted to evaluate news coverage of food assistance using an equity lens during the second year of the pandemic, a period when policy changes occurred at the state and federal levels to address food insecurity and accessibility to safety net programs. For example, states implemented school lunch waivers, which provided food for all children regardless of their household income, though many states ultimately allowed the program to expire as the pandemic wore on.^6^

During the second year of the pandemic, there were also institutional changes in the design and delivery of food assistance programs that increased flexibility for participants and streamlined programs.^7–9^ Such programmatic changes helped to address existing inequities in food security. For example, people of color were disproportionately affected by food insecurity during the pandemic. Approximately 4 in 10 families with Hispanic/Latinx parents (39.1%) and Black parents (40.8%) reported food insecurity—almost triple the rate of families with White parents (15.1%).^10^

Our current analysis focuses on how, if at all, news coverage of food assistance framed racial and health equity, and the possible implications of this framing for narrative change going forward. We assessed whether equity was explicitly named in coverage of food assistance policies, and specifically examined whether a racial equity lens was present in reporting on these issues.

## Methods

We conducted an ethnographic content analysis^11^ to examine print news articles. Owing to concerns of misinformation and fake news, we limited our analysis to outlets validated by the LexisNexus selection process.^12^ We searched the LexisNexis database for articles about food insecurity or food assistance published by U.S. newspapers and wire services between January 1, 2021, and December 31, 2021. We developed a search string to capture mentions of food assistance programming such as general food assistance, Supplemental Nutrition Assistance Program, school lunches, and Special Supplemental Nutrition Program for Women, Infants, and Children.

We retrieved 1782 articles from LexisNexis, then used two constructed weeks^13^ to gather a representative sample of 323 articles. Constructed week sampling is a method of sampling for content analysis that is preferred to random sampling because it accounts for variation of news content over a 7-day news week.^14^

Our final sample for in-depth coding included 298 relevant articles. We designed a coding instrument modified from our previous analysis^4^ to capture patterns in news coverage including mentions of (1) types of food assistance, (2) safety net assistance for different demographic groups, and (3) arguments for providing food assistance that evoked or directly named equity as a rationale, or otherwise evoked equity. We held discussions to define equity:^15^ based on those conversations we searched each article for phrases such as “benefits everyone,” “evens the playing field,” “everyone deserves access to,” “combats disparities,” in addition to the terms “equity” and “equitable.”

We performed intercoder reliability testing to ensure that coder alignment did not occur by chance. We achieved satisfactory reliability measures for certain coding variables (Krippendorff's *α*>0.8)^16^: for variables where we did not achieve inter-coder reliability we held consensus conversations supported by qualitative notes recorded during coding.

## Findings

During the second year of the pandemic, U.S. print news highlighted the importance of expanding and sustaining food assistance programs for children and families. For example, 56% of articles specifically mentioned “kids, youth, or children,” 81% of articles mentioned “families,” and 29% specifically mentioned K-12 students. In articles wherein people spoke about their experiences receiving food assistance (*n*=37), the majority of speakers quoted (68%) were parents seeking assistance for their families.

Just 28% of articles argued that food assistance programs are justified because they help address inequities in food access: other arguments for expanding assistance focused on improved nutrition and personal health or benefits to the economy. When arguments about equity appeared, they were most often in discussions of school lunch programs, as in an article that called for universal free school meals for K-12 students because the practice “reduces shame around hunger by keeping everyone on a level playing field.”^17^

When equity arguments did appear, they rarely focused on racial inequities in food assistance, or solutions to address them. Only 6% of articles mentioned people of color as recipients of food assistance programs, and even fewer (4%) specifically evoked addressing racial inequity as a rationale for program change. A rare article that framed food assistance in the context of racial equity included a quote from the director of the Center for Hunger-Free Communities at Drexel University, who noted “there are major problems with the way we treat children and the way we treat Black and Latino families in America. We fail to protect those who need the most protecting.”^18^

Although over half of articles mentioned changes to the delivery and design of food assistance programs implemented to reduce barriers to access, these changes were seldom framed as strategies to address inequities. A rare article noted that “programs are hindered by inadequate staffing and technology simply because the poor people they serve lack political clout,” and included remarks from scholars arguing that “excessive bureaucracy deepens poverty and inequality.”^19^

## Discussion

Our analysis of news about food assistance during the second year of the COVID-19 pandemic found that, as in our previous analysis, news coverage failed to connect the programs to achieving health equity. Equity arguments focused on the needs of children and families, usually in the context of the school lunch program, but there was little mention of equity around other programmatic changes, nor mention of food assistance programs as a way to address racial inequities even though people of color suffer disproportionately from food insecurity.^20^

Such “color blind” reporting about food assistance programs may reflect the fact that many policies around food assistance programs fail to explicitly name racial disparities. Such policy solutions may be seen as “universal” in their impact,^21^ but could fail to help those who are most marginalized.

One way to reframe universal policies is through “targeted universalism.”^21,22^ Targeted universalism calls for setting universal goals, while establishing targeted processes to achieve those goals that first help those most marginalized or experiencing inequity. Although journalists and editors make many of the final decisions, advocates and researchers have the opportunity to help shape the news narrative through interviews and opinion pieces. They can use these opportunities to frame food assistance policies in the context of targeted universalism and explicitly name how these programs help address inequities to help expand understanding of these policies, deepen their appeal in key communities, and broaden public perceptions around who deserves to receive support.

Our assessment is limited because we evaluated only a sample of articles published between January 1, 2021, and December 31, 2021, which may not reflect the entirety of the debate around food assistance policy during this phase of the COVID-19 pandemic; however, our constructed week sampling helps assure that the coverage we assessed was representative. We also did not assess photographs associated with the articles, which may have conveyed additional cues about race. Finally, we were unable to directly compare equity arguments with findings from our previous article^4^ because we did not explicitly code for equity language in that analysis. Future research could explore temporal trends in equity framing in news about food assistance and other safety net programs.

## Health Equity Implications

Food assistance programs are longstanding efforts to remediate inequities and provide “a critical nutrition and hunger safety net,” especially for children.^23^ Even so, government food assistance remains controversial. News coverage is not doing all it could to enrich the discourse around food assistance: although marginalized groups were disproportionately impacted by the pandemic, the news media rarely framed food assistance as a way to address racial equities. Reframing coverage could expand the discourse around food assistance to explicitly depict food assistance and other safety net programs as tools to advance racial equity.
